# On the Fair Division of Multiple Stochastic Pies to Multiple Agents within the Nash Bargaining Solution

**DOI:** 10.1371/journal.pone.0044535

**Published:** 2012-09-14

**Authors:** Athanasios C. Karmperis, Konstantinos Aravossis, Ilias P. Tatsiopoulos, Anastasios Sotirchos

**Affiliations:** 1 Sector of Industrial Management and Operational Research, School of Mechanical Engineering, National Technical University of Athens, Athens, Greece; 2 Army Corps of Engineers, Hellenic Army General Staff, Ministry of Defence, Athens, Greece; Cinvestav-Merida, Mexico

## Abstract

The fair division of a surplus is one of the most widely examined problems. This paper focuses on bargaining problems with fixed disagreement payoffs where risk-neutral agents have reached an agreement that is the Nash-bargaining solution (NBS). We consider a stochastic environment, in which the overall return consists of multiple pies with uncertain sizes and we examine how these pies can be allocated with fairness among agents. Specifically, fairness is based on the Aristotle’s maxim: “*equals should be treated equally and unequals unequally, in proportion to the relevant inequality*”. In this context, fairness is achieved when all the individual stochastic surplus shares which are allocated to agents are distributed in proportion to the NBS. We introduce a novel algorithm, which can be used to compute the ratio of each pie that should be allocated to each agent, in order to ensure fairness within a symmetric or asymmetric NBS.

## Introduction

Over the last decades, effective management of cooperative structures has been of great interest, due to its application to various real life situations. Generally, in all kinds of cooperative structures there is a nonnegative surplus included in the overall return, which may consist of more than one pie. For instance, in a supply chain network with multiple production facilities that produce different products, the manufacturing and distribution costs as well as the unit profits are dissimilar. Another example is a water/power/waste management system that commonly includes the collection and distribution of water/power/waste arising from different sources. In most cases, these multiple pies should be shared among a finite set of agents who receive specific payoffs if they disagree to cooperate, i.e. through the non-cooperative option. Moreover, since a cooperative venture is formed before the actual size of each pie is realized, these pies can be assumed as stochastic variables, i.e. pies with uncertain sizes. In such a stochastic environment with multiple pies and multiple agents, main challenge is to ensure fairness, especially when risk-neutral agents have already reached an agreement for the division of the overall surplus. The subsequent sections of this paper include the review of the related literature, the description of the basic problem, the introduction of a novel computation method and the discussion of possible applications and future research issues.

### Solution Concepts in Cooperative Games

More than 2000 years ago, Aristotle in “Nicomachean Ethics” has established the main principle of fairness. He indicates that equals must be treated equally and unequals must be treated unequally, in proportion to relevant similarities and differences [1], [2]. This is a formal principle, which has been applied to all kind of cooperative structures, e.g. equal treatment and proportionality are two general principles of European Union Law.

Cooperative game theory is applied to a finite set of agents, namely grand-coalition, while any subset in which this set can be divided is called coalition [3], and a coalition with just one agent is called singleton. A cooperative game for a grand-coalition *N* = {1,2,3,…,*n*}, is either a pair (*N*, *p*) and a characteristic function *p* : 2*^N^* → ℜ, with *p*(Ø) = 0, which represents the collective payoff for a set of agents forming a coalition [4], or a pair (*N*, *c*) and a characteristic function *c* : 2*^N^* → ℜ, with *c*(Ø) = 0 that describes the cost for a set of agents who cooperate in accomplishing a specific task [5]. The solution of the game is a vector *x* Є ℜ*^N^* representing the allocation of the overall profit *p*(*N*) or cost *c*(*N*) to each agent. In general, a nonnegative surplus can be shared with fairness following the equal or proportional sharing methods [6]. In the literature, several papers aim to provide axiomatic characterizations of fair solutions, while a different approach of fairness results in a different solution concept. These are the solution of von Neumann–Morgenstern [7], the Shapley value [8], the core [9], and the Nucleolus [10]. Moreover, alternative axioms are discussed in [6], [11], [12], [13]. However, the most widely applied solution for the cooperative bargaining problem with fixed disagreement payoffs is the Nash-bargaining solution (NBS) [14]. The NBS consists of an axiomatic derivation of the solution for a bargaining game between two agents, who have perfect information [15], [16] and examine to cooperate and share a specific surplus. This solution, which can be easily expanded in more than two agents [17], [18], satisfies a set of axioms: linear invariance and independence of irrelevant alternatives (the solution is preserved under the monotone transformation of the agents’ utility functions or the exclusion of non-selected alternatives from the bargaining set); Pareto-optimality (any change to a different allocation that makes one agent better off will make the other agent worse off); feasibility (the sum of the agents’ allocations does not exceed the shared pie) and symmetry (identical agents receive equal utility allocations). Moreover, the asymmetric NBS is applied to cases with non-identical agents [19], [20].

### Surplus Sharing Mechanisms and Bargaining in Stochastic Environment

In recent papers, the fair division of a surplus among risk-averse agents is examined in [21] and several authors analyze the profit-sharing [22], revenue-sharing [23], cost-sharing [24] and cost-revenue sharing [25], [26] mechanisms in cooperative structures. Other surplus division models are applied to supply network formation [27], to decentralized supply chains [28], [29], and to river water [30] and groundwater [31] sharing cases. However, taking into consideration that a surplus sharing mechanism should use risk as driver [32], there is a strong interaction between the divisions of a surplus with the risk allocated to agents. For instance, in [33] is developed an analytic model based on the maximization of the probability that a firm will achieve some given profit target, and in [34] is developed a risk-based process that can be used to compute the range including the win-win funding schemes of partnerships at a predefined level of probability.

As mentioned in [35], the theoretical literature on bargaining in stochastic environment is limited. In particular, few papers examine cases where agents are negotiating over a surplus with uncertain size, i.e. they receive individual surplus shares (called dividends) with uncertain sizes. One of the mostly used techniques for handling uncertainty is the Monte Carlo Simulation (MCS), which takes into account the impact of a set of stochastic variables (inputs) and defines the possible range of the output values graphically expressed as the cumulative probability distribution function [36], [37], [38], [39].

However, a rational investor would like to minimize the probability of the expected losses [40], or/and to maximize the probability of the expected gains. This implies that an allocation with which the probability of being negative an agent’s dividend is high and the probabilities of being negative the opponents’ dividends are low, cannot be considered as fair. In other words, a fair surplus division has to be based not only on the expected values of the agents’ dividends, but also should take into account their standard deviations. If agents agree in a specific sharing scheme of the overall surplus within the NBS, then fairness is achieved when the mean values and the standard deviations of the agents’ dividends are proportional to the NBS. Proportionality implies that when the surplus is divided equally, the expected dividends and their standard deviations should be equal [26]. Specifically, in contrast with [41], which indicates the impossibility of fair risk allocation, in [26] is developed a basic model for the equal profit and risk allocation among agents who examine to cooperate by undertaking parts of the system cost individually and share the remaining costs and revenues. It is proved that when two pies with uncertain sizes are allocated to non-identical agents, there is a finite set of possible solutions that depends on the number of agents. However, this model is limited in cases with two stochastic pies. Herein, we consider a stochastic environment, in which the overall return consists of multiple pies with uncertain sizes and the surplus is not necessarily divided equally, i.e. when agents negotiate and agree in a specific NBS, this solution can be either symmetric with equal payoffs or asymmetric with equal or unequal payoffs [19], [20], [42]. The main objective of this paper is to introduce a novel method that can be used to compute the ratio of each pie that should be allocated to each agent, in order to ensure fairness within a specific NBS. A complete list of the notations used in this paper is presented in Table 1 and the proofs of Theorems and Propositions are shown in Supporting Information Text S1.

**Table 1:** List of notations.

Description	Symbol
Finite set of *n* agents (grand-coalition)	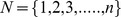
Finite set of *m* stochastic variables (pie-set)	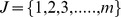
Disagreement payoff of agent *i* (if agents do not reach an agreement, then their payoff vector is : (C_1_,C_2_, …,C_n_)	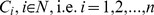
Stochastic (random) variable *j* with a normal probability distribution: (where σ *^j^*>0, μ *^j^*>0 for pies representing gains and μ *^j^*<0 for pies representing losses)	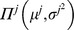
Grand-coalition’s overall return	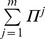
Surplus to be divided	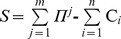
Ratio of pie *j* which is allocated to agent *i*	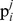
Efficient allocation of all pies	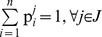
Stochastic individual surplus share (dividend) allocated to agent *i*	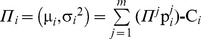
Expected value of the dividend allocated to agent *i*	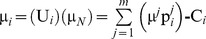
Standard deviation of *i* ’s dividend	σ *_i_*
Nash-bargaining solution U Є **R** *^N^*	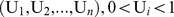
Set of coalitions including agent *i*, which arise through a specific partition set of the grand-coalition *N* into two nonempty coalitions for *n*-1 times.	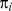
Ratio of subset of pies {1,.,g}, which are allocated to agent *i*	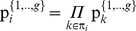
Characteristic function	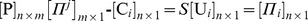

### Problem Description and Mathematic Formulation

#### Assumptions

We consider a finite number of agents indexed by *i*. Let *N* = {1,2,3,…,*n*} denote the grand-coalition of all agents. The following assumptions are used throughout the paper:

• **Assumption 1.** There is complete information among agents, who examine the cooperative option having specific disagreement payoffs. This implies that if they do not reach an agreement, then their payoff vector is : (C_1_,C_2_, …,C*_n_*). In this case, the participants’ objectives are partially cooperative, as they aim at reaching an agreement and partially conflicting, because each agent has its own utility function regarding the negotiation outcome [43].
•** Assumption 2.** The different gains and losses that are yielded through cooperation forming a finite set of pies *J* = {1,2,3,…,*m*} that is called pie-set. These pies are divisible and should be shared among agents. However, since the grand-coalition is formed before the actual size of each pie is realized, all pies are assumed to be stochastic variables [44]. Specifically, all pies indexed by *j* follow normal probability distribution functions with specific mean values and variances: 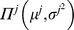, where σ*^j^*>0 for all pies, µ*^j^*>0 for pies representing gains and µ*^j^*<0 for pies representing losses.
•** Assumption 3.** Agents are rational, i.e. each agent should get at least as much as it could obtain through the non-cooperative option. Clearly, the cooperation yields a nonnegative surplus *S*. That is, the *N*’s overall return 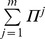
is equal to the sum of agents’ disagreement payoffs 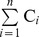 plus the surplus *S* [6]: 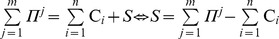

•** Assumption 4.** All agents are risk-neutrals, i.e. they are indifferent between the *m* pies, since they consider only the overall expected return when making investment decisions [38]. In particular, they negotiate over the division of the surplus that is yielded through cooperation and the bargaining outcome is the NBS, which is a vector: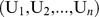 representing the expected individual surplus shares (dividends) which are allocated to agents. This is the unique solution that maximizes a function equal to the product of the agents’ utility net gains from cooperation, measured relative to the exogenous disagreement outcome [17], [18], [45].

#### Axioms

The allocation of the surplus *S* that is yielded through cooperation fulfils the following axiom:

•* Coalitionally rational* [46]: This axiom is satisfied when the expected return (sum of expected gains minus the relative losses) is greater than the sum of agents’ disagreement payoffs, i.e. the *S*’s mean value, denoted by µ*_N_*, should be positive:

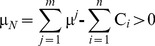
(1)Since all agents are risk-neutrals, they negotiate over the *S*’s mean value considering their expected dividends (µ*_i_*). The bargaining outcome is an allocation, i.e. a vector U Є ℜ*^N^* representing the ratio of the µ*_N_* that is divided in agent *i* Є *N*:
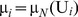
(2)


All agents are assumed to be rational and thus the allocation U Є ℜ*^N^* fulfils the following axiom:

•* Individually rational*: Agent *i* get at least as much as it could obtain through the non-cooperative option, according to the following inequality:

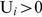
(3)•* Linear Invariance and Independence of Irrelevant Alternatives*: Since the bargaining outcome is assumed to be the NBS, it has to be linear invariance and independent of irrelevant alternatives. Let *F* be the feasible set of allocations. If *F* ‘is obtained from *F* by multiplying all agents’ utilities by α*_i_*, then the solution of the new game is obtained by multiplying each agent’s coordinate in the first solution by α*_i_*. Moreover, the solution remains the same for each subset of *F* that includes the specific solution.
•* Feasibility and Pareto-Optimality*: In order to fulfill the feasibility and Pareto-optimality axioms, the allocation should be efficient, i.e. the sum of expected dividends equals the expected surplus:

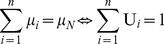
(4)Eq (4) ensures feasibility, as the sum of allocations does not exceed the overall surplus. Moreover, inequality (3) and Eq (4) ensure Pareto-optimality, because for any other allocation U’≠ U, with which at least one agent *i* is better off: U*_i_*’>U*_i_*, and from Eq (2): µ*_i_’*>µ*_i_*, there will be at least one agent *k* worse off: U*_k_*’<U*_k_*, and from Eq (2): µ*_k_’*<µ*_k_*. Hence, there are no Pareto improvements which can be made in U and the negotiation result is Pareto-Optimal.

Let
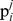
denote the ratio of pie *j* Є *J* which is allocated to agent *i* Є *N.* It is clear that agent’s *i* expected dividend is: 
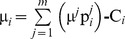
, while her expected overall return is: 
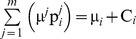
. Due to the fact that an inefficient allocation of at least one pie *j* will leave space for renegotiation, we consider the efficient allocation of all pies *j* Є *J*,:
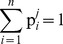
(5)

•* Symmetry*: On one hand, if all agents are identical (equal disagreement payoffs and symmetric utility functions), then they will agree in the equal allocation of the overall surplus, according to Eq (6):

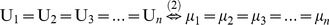
(6)On the other hand, if agents are non-identical, then the bargaining outcome will be the asymmetric NBS [19]. In the asymmetric NBS, the surplus can be divided either equally fulfilling Eq. (6) [42], or unequally with U*_i_* ≠ U*_k_* for at least two agents *i*, *k* Є *N*.

#### The axiom of fair division

In cases where multiple pies should be shared among multiple agents within a symmetric or asymmetric NBS, main challenge is to ensure fairness for the division of the overall surplus [47]. Let *Π_i_* denote the individual stochastic surplus share, which is allocated to agent *i*. This is given from Eq (7):
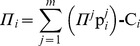
(7)


Taking into consideration that all pies *j* Є *J* are normally distributed and the agents’ disagreement payoffs C*_i_* are real numbers, we conclude that the stochastic dividends which are allocated to agents follow normal probability distribution functions: 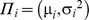. Moreover, a solution satisfying U consists of the ratios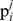 for all *i* Є *N* and *j* Є *J.* In particular, a solution can be illustrated in a [P]*_n_*_X*m*_ matrix, in which the 1,2,.,*n* rows denote the agents and the 1,2,.,*m* columns denote the pies, i.e. each element
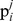
represents the ratio of pie *j* which is allocated to agent *i*:
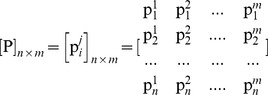
(8)


In this case, the characteristic function of the dividends allocated to all agents within a symmetric or asymmetric NBS can be expressed in Eq (9):
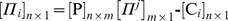
(9)


However, fairness is achieved when all these dividends are distributed in proportion to the NBS: 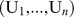, i.e. the expected values of the dividends which are allocated to agents should satisfy:
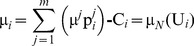
(10)and the dividends’ standard deviations, denoted by σ*_i_*, should be also proportional to the mean values, according to Eq (11):



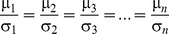
(11)In other words, fairness is achieved when the dividends 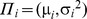 which are allocated to all agents are distributed in proportion to the NBS, fulfilling Eq (12):
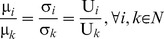
(12)


## Results

Even though most papers dealing with cooperative games use a bottom-up approach examining which coalition of agents can be formed, or how sub-coalitional gains can be allocated in order to secure a sustainable agreement, herein we follow a top-down approach.

### Computing Solutions for Fair Surplus Division

**Figure 1:**
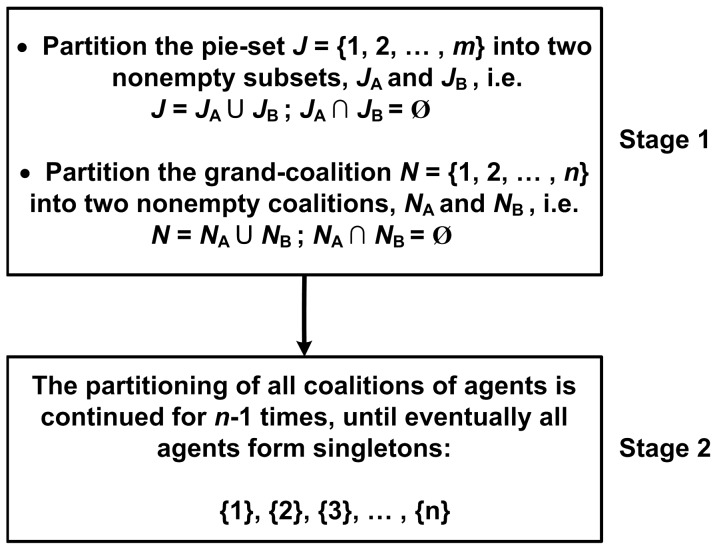
A general method for fair surplus division.

A novel approach to compute the ratio of each pie that should be allocated to each agent, in order to ensure that the surplus is divided in proportion to the NBS, is presented in Figure 1. As can be seen, the general method introduced with this paper includes two basic stages, which are following analyzed:

#### Stage 1: Partition the pie-set *J* into two nonempty subsets: *J_A_*, *J_B_*, and the grand-coalition N into two nonempty coalitions *N_A_ N_B_*

Initially, we consider that we have to use the above Eqs (1) to (10), in order to estimate the (*n*×*m*) unknowns of a [P]*_n_*_X*m*_ matrix that satisfy the axiom of fairness. However, through the partition of the pie-set *J* = {1,2,.,*m*} into two subsets: *J*_A_ = {1,…,*g*}, and *J*_B_ = {*g*+1,…,*m*}, with 1≤ *g*<*m*, the characteristic function is presented in Eq (13):
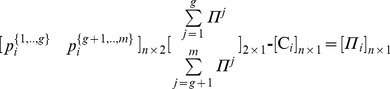
(13)where
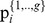
denote the ratio of the subset *J*_A_ = {1,…,*g*} allocated to agent *i*, and
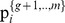
denote the ratio of the subset *J*_B_ = {*g*+1,…,*m*} allocated to agent *i*. This implies that the ratios of pie 1, pie 2, …, and pie *g*, which are allocated to agent *i* are equal to: 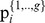, and the ratios of pie *g*+1, pie *g*+2, …, and pie *m*, which are also allocated to agent *i* are equal to: 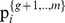.

Moreover, through the partition of the grand-coalition *N* into two coalitions: *N*_A_ = {1,…, *h*}, *N*_B_ = {*h*+1,…,*n*}, with 1≤ *h*<*n*, we have 4 unknowns, which are the ratios of each subset: *J*_A_ = {1,…,*g*} and *J*_B_ = {*g*+1,…,*m*} allocated to each coalition: *N*_A_ = {1,…, *h*} and *N*_B_ = {*h*+1,…,*n*}:
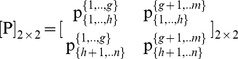
(14)


Specifically, through the partitions of the pie-set and the grand-coalition into a pair of two nonempty subsets and two nonempty coalitions, the characteristic function (9) is expressed in Eq (15):
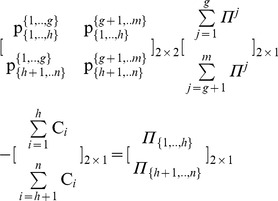
(15)


Further, we derive the following Theorem 1.

#### Theorem 1

*For each pair of two coalitions and two subsets that can arise from the partition of the grand-coalition* N = {1,.,n} into: N_A = _{1,.,h}and N_B = _{h+1,.,n}, (N_A_ U N_B_ = N; N_A_ ∩ N_B_ = Ø) *and the partition of the pie-set* J = {1,.,m} into: J_A = _{1,.,g} and J_B = _{g+1,.,m}, (J_A_ U J_B_ = J; J_A_ ∩ J_B_ = Ø), *there is a unique* [P] _2×2_ *matrix:* 
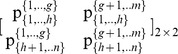
, *which ensures fairness within the NBS satisfying Eq (12):* 
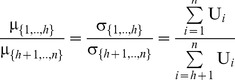


#### Stage 2: Continuous partitions of the agents’ coalitions for *n*-1 times

From Theorem 1, it is clear that through the partition of the coalition *N*_A_ = {1,.,*h*} into two nonempty coalitions {1,., *f*} and {*f*+1,…,*h*}, 1≤ *f*<*h*, Eq (15) gives:
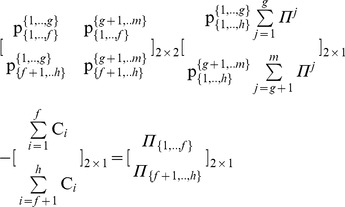
(16)which has a unique [P] _2×2_ matrix: 
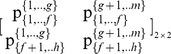
satisfying Eq (12) and 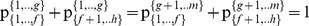.

Similarly, through the partition of the coalition *N*_B_ = {*h*+1,.,*n*} in another pair of nonempty coalitions {*h*+1,., *k*} and {*k*+1,…,*n*}, *h*+1≤ *k*<*n*, Eq (15) gives:
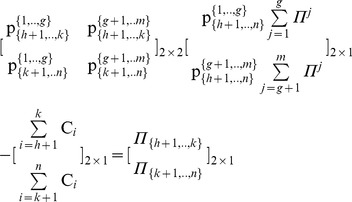
(17)which also has a unique [P] _2×2_ matrix: 
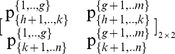
 satisfying Eq (12) and 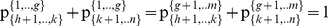.

In particular, the partitioning of coalitions into two nonempty coalitions is continued, until eventually all agents form singletons: {1}, {2}, {3},…, {*n*}, i.e. for *n*-1 times. Through this process, if we compute the unique [P] _2×2_ matrix for each coalition, we can compute the ratio of each subset {1,.,*g*} and {*g*+1,.,*m*} which is allocated to each agent.

Let 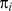 denote the set of coalitions including agent *i*, which arise from a specific set of partitions of the grand-coalition *N* into two nonempty coalitions for *n*-1 times. For instance, through the continuous partitions of the *N* = {1,2,3,4,5} within a specific partition set, into: {1} and {2,3,4,5}, and the further partition into {3} and {2,4,5}, and the further partition into {2} and {4,5} and the further partition into {4} and {5}, the sets of coalitions 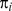
including agents are:
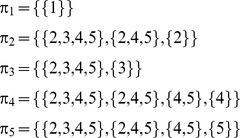



That is, the ratio of each subset of pies ({1,.,*g*} and {*g*+1,.,*m*}), which is allocated to agent *i*, equals the product of ratios of the coalitions, in which *i* is included, e.g. for agent 5:
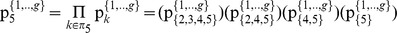


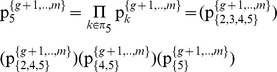



However, the ratio for agent *i* in each subset of pies {1,…,*g*} and {*g*+1,…,*m*}, equals her ratios in all pies of the specific subset: 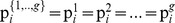
and 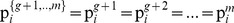.

In other words, through the partition of the pie-set *J* into two nonempty subsets, and the continuous partitions of all coalitions into a pair of nonempty coalitions for *n*-1 times (until all agents form singletons), we can compute the ratios 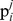 of all pies *j* = 1,2,.,*m* which are allocated to all agents *i* = 1,2,.,*n*. That is, we can compute a specific matrix [P] *_n_*_×*m*_, which ensures that the agents’ dividends 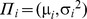 are distributed in proportion to the NBS: 
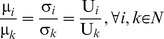


### Basic Features

In this section, we present some basic features of the proposed method.

#### Number of possible partitions of the pie-set

Let *g*(*m*) denote the possible combinations for the partition of the pie-set *J* into two nonempty subsets. We derive the following Proposition 1.

#### Proposition 1

*The number of possible partitions of a pie-set J into two nonempty subsets is given from the piecewise Eq (18):*

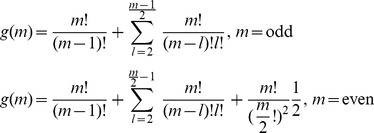
(18)


#### Finite possible [P] *_n_*_×*m*_ matrices for fair surplus division

However, in each partition of the grand-coalition *N* into two nonempty coalitions according to the first stage of the proposed method, or any other partition of the agents’ coalitions into two nonempty coalitions according to the second stage, there is no coalition that can be profitably blocked. This implies that there is no constraint considered and any agent can be placed either in the first or the second coalition of each partition, in which the order of agents does not matter. Let *f*(*n*) denote the possible combinations for the continuous partitions of coalitions for *n*-1 times (until all agents form singletons: {1},…,{*n*}. Taking into consideration that for each partition into two nonempty coalitions there is a unique [P] _2×2_ matrix, we derive Theorem 2.

#### Theorem 2

*The number of possible* [P] _n×m_ *matrices that ensure fairness for the surplus division within a NBS, is finite and equals the product of the possible partitions of the pie-set into two subsets with the possible partitions of all coalitions of agents into two nonempty coalitions for n-1 times:*

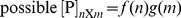
(19)where:



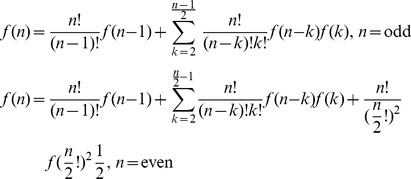
(19.1)

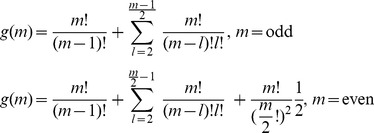
(19.2)


#### Possible [P] *_n_*_×*m*_ matrices for 2*≤ n*, *m ≤*10

The precise number of possible [P] *_n_*_×*m*_ matrices for 2≤ *m*, *n* ≤10, is illustrated in Table 2. As can be seen, this Table presents the numbers of possible matrices for all combinations of: *m*, *n* = 2,3,4,., 10. For instance, when there are 7 stochastic pies to be allocated to 5 agents, there are 6615 matrices [P] _5×7_ satisfying Eq (12), and when there are 5 stochastic pies to be allocated to 7 agents, there are 155925 matrices [P] _7×5_ satisfying Eq (12).

**Table 2:** Number of possible [P] *_n_*_×*m*_ matrices for fair surplus division.

		Number of pies								
		*m* = 2	*m* = 3	*m* = 4	*m* = 5	*m* = 6	*m* = 7	*m* = 8	*m* = 9	*m* = 10
Number of Agents	*n* = 2	1	3	7	15	31	63	127	255	511
	*n* = 3	3	9	21	45	93	189	381	765	1533
	*n* = 4	15	45	105	225	465	945	1905	3825	7665
	*n* = 5	105	315	735	1575	3255	6615	13335	26775	53655
	*n* = 6	945	2835	6615	14175	29295	59535	120015	240975	482895
	*n* = 7	10395	31185	72765	155925	322245	654885	1320165	2650725	5311845
	*n* = 8	135135	405405	945945	2027025	4189185	8513505	17162145	34459425	69053985
	*n* = 9	2027025	6081075	14189175	30405375	62837775	127702575	257432175	516891375	1035809775
	*n* = 10	34429425	103378275	241215975	516891375	1068242175	2170943775	4376346975	8787153375	17608766175

### Computation Algorithm

In this section we introduce an algorithm, which can be used to compute a [P] *_n_*_×*m*_ matrix that ensures fairness for the division of the overall surplus. This algorithm consists of seven basic steps, which are presented below:

1 Step 1: Randomly partition the pie-set *J* into two subsets: {1,…,*g*} and {*g*+1,…,*m*} with 1≤ *g*<*m*.
2 Step 2: Randomly partition the grand-coalition *N* into two coalitions: {1,…,*h*} and {*h*+1,…,*n*} with 1≤ *h*<*n*.
3 Step 3: Develop a MCS model, in which the C*_i_*, *Π ^j^* are inputs and the *Π*_{1,.,*h*}_, *Π*_{*h*+1,.,*n*}_ the outputs, according to the following Eqs (20) and (21):

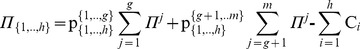
(20)

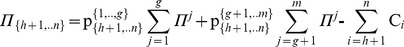
(21)

4 Step 4: Select a specific value of 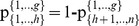, and estimate the 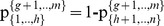, in order to fulfill:

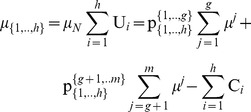
(22)

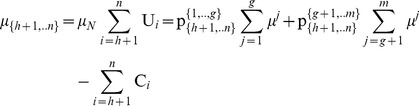
(23)


For the 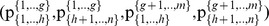 that fulfils Eqs (22), (23) run the MCS and estimate σ_{1,.,*h*}_, σ_{*h*+1,.,*n*}_. If the following Eq (24) is fulfilled then go the next Step, otherwise examine alternative values of 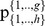 *(*simply by increasing/decreasing its initial value), until you find the unique elements of a [*P*]_ 2×2_ matrix: 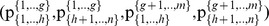, which fulfils Eq (24):
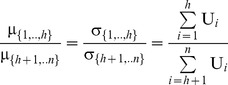
(24)

5 Step 5: Use the 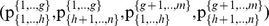, and return to Step 2, i.e. randomly partition both the {1,…,*h*} and {*h*+1,…,*n*} coalitions into two pairs of nonempty coalitions: {1,…,*f*}, {*f*+1,…,*h*} and {*h*+1,…,*k*}, {*k*+1,…,*n*}, respectively, and compute the unique ratios: 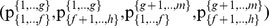 and 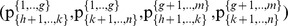. Specifically, the 2 to 5 Steps should be followed for *n*-1 times.
6 Step 6: Calculate the ratio of each subset {1,…,*g*} and {*g*+1,…,*m*} allocated to each agent, through the product of the ratios of the agent-coalitions in which the agent is included. Moreover, due to the fact that each agent’s ratios are equal in all pies of each subset, illustrate the [P] *_n_*_×*m*_ matrix.
7 Step 7: In order to verify the results, develop another MCS model, in which the computed [P] *_n_*_×*m*_, the [*Π ^j^*] *_m_*_×1_, and the [C*_i_*] *_n_*_×1_ matrices are inputs, and the [*Π _i_*] *_n_*_×1_ is the output according to Eq (9):

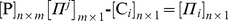
(9)Run the simulation and estimate: 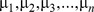, and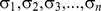, in order to verify that the dividends are allocated to all agents with fairness: 
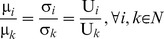


## Discussion

### Numerical Example

In this section we present a numerical example with the application of the proposed computation algorithm. We consider 4 risk-neutral agents forming a grand-coalition *N = *{1, 2, 3, 4}. All agents are rational and agree to cooperate having different disagreement payoffs, i.e. through the non-cooperative option they receive: C_1_ = 2×10^6^, C_2_ = 5×10^6^, C_3_ = 3×10^6^, and C_4_ = 2×10^6^. We examine a general case with non-identical agents and the asymmetric NBS, considering that the symmetric NBS can be included as a special case where agents’ utility functions are symmetric and the disagreement payoffs are equal: C_1_ = C_2_ = C_3_ = C_4_. There are 5 different pies representing the *N*’s gains through cooperation, i.e. the pie-set is: *J* = {1, 2, 3, 4, 5}. Since the grand-coalition is formed before these gains are realized, each pie indexed by *j* follows a normal probability distribution function with specific mean value (μ *^j^*) and standard deviation (σ *^j^*), as presented in Table 3. All agents are risk-neutrals implying that they are indifferent between the *m* pies, as they consider only their expected dividends. Specifically, they negotiate over the expected value of the surplus (μ*_Ν_*), which is yielded through cooperation. This value is computed with Eq (1):
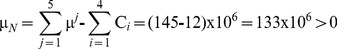
(1)


**Table 3:** Normal probability distributions of pies (values×10^6^).

Pies	*j* = 1	*j* = 2	*j* = 3	*j* = 4	*j* = 5
Mean values: μ *^j^*	15.0	20.0	50.0	10.0	50.0
Standard deviations: σ *^j^*	4.0	2.5	2.0	4.0	2.0

The bargaining outcome is a vector U Є ℜ*^N^* representing the ratio of the µ*_N_* that is allocated to agent *i*. The subsequent sections examine two cases within the asymmetric NBS, which is the payoff that maximizes a weighted product of players' gains over their disagreement payoff.

#### Asymmetric NBS with unequal divisions

The first case is the asymmetric NBS where the surplus is divided unequally among agents. We assume that this solution is: U = (U_1_, U_2_, U_3_, U_4_) = (0.12, 0.25, 0.30, 0.33). Since all pies included in Table 3 should be allocated to all agents with fairness, we want to compute the ratio of each pie that should be allocated to each agent, in order to ensure that the agents’ dividends [Π*_i_*]_ 4×1_ are distributed in proportion to the NBS, i.e. to compute a [P]_ 4×5_ matrix, which satisfies Eq (12): 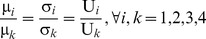


**Figure 2:**
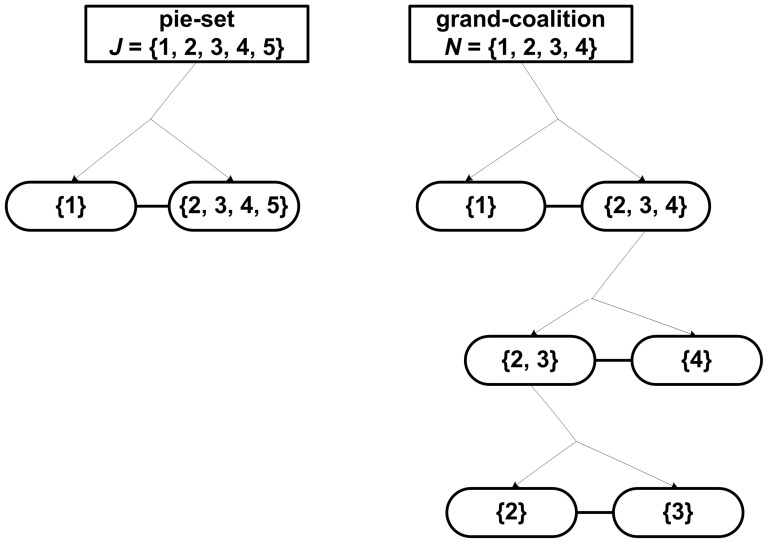
First set of partitions.

We use the computation algorithm presented in the previous section, i.e. the random partition of the pie-set into two subsets and the continuous random partitions of the agent-coalitions for *n*-1 times, as illustrated in Figure 2.

1 Step 1: Randomly partition of the pie-set *J* into two subsets: {1}, and {2, 3, 4, 5}.
2 Step 2: Randomly partition of the grand-coalition *N* into two coalitions: {1}, and {2, 3, 4}.
3 Step 3: A MCS model is developed, in which the C*_i_*, *Π ^j^* are inputs and the *Π*_{1}_, *Π*_{2,3,4}_ are the outputs, according to the following Eqs (25), (26):

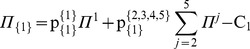
(25)

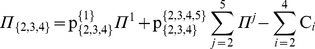
(26)

4 Step 4: Initially, we select a specific value: 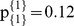. For this value, the: 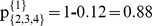, while from Eqs (22), (23), we compute: 
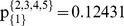
 and the 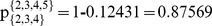:

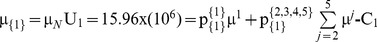
(27)

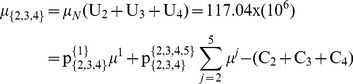
(28)
  For the scenario 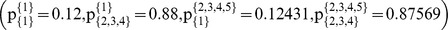, we run the MCS and we get: σ_{1}_ = 6232764, σ_{2,3,4}_ = 873019. It is clear that this scenario does not satisfy Eq (24), because:
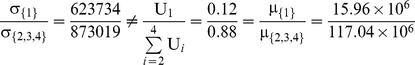

  Further, we examine alternative scenarios arising from different values of 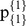, and we compute the unique ratios that satisfy Eq (24):
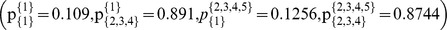

5 Step 5: We return to Step 2 and use the ratios: 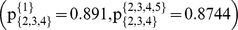 in the random partition of the {2, 3, 4} coalition into two coalitions: {2,3} and {4}. According to this partition, we compute the unique ratios: 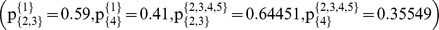. Further, we return to Step 2 and we use the ratios 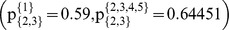 in the partition of the coalition {2, 3} into {2} and {3}, in order to calculate the unique ratios: 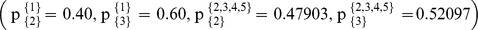

6 Step 6: We estimate for each agent the ratios in each subsets of pies: {1}, and {2, 3, 4, 5}, through the product of the ratios of the agent-coalitions in which the agent is included:

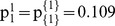
and 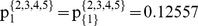

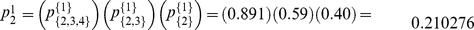

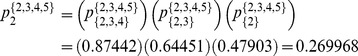

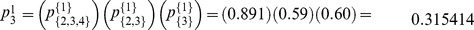
 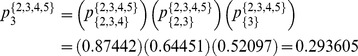

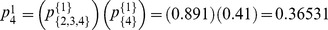
 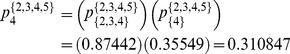

Moreover, due to the fact that each agent’s ratios are equal in all pies of each subset, we estimate:

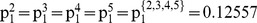

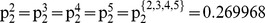

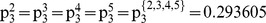

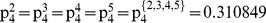

and we illustrate the [P] _4×5_ matrix:



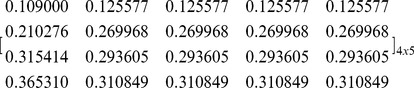
(29)(7) Step 7: In order to verify the results, we develop another MCS model, where the estimated [P] _4×5_, the [Π *^j^*] _5×1_, and the [C *_i_*] _4×1_ matrices are inputs, while the [Π *_i_*] _4×1_ is the output according to Eq (9):



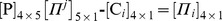
(9)We run the simulation that gives the following results:


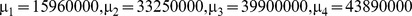
and 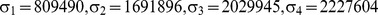. These results verify that the overall surplus is divided with fairness, as the dividends allocated to all agents are distributed in proportion to the NBS:
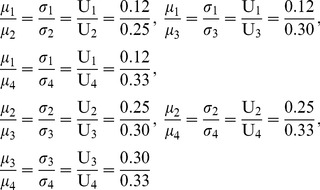
(30)


#### Other solutions for asymmetric NBS with unequal divisions

**Figure 3:**
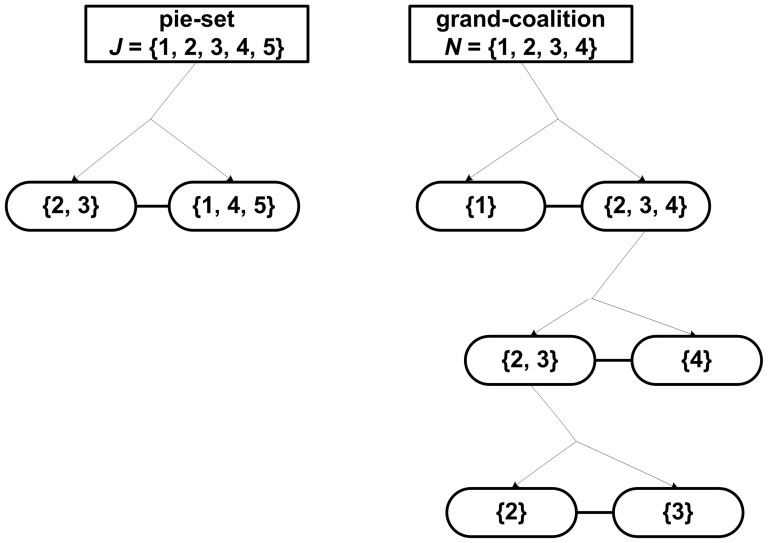
Second set of partitions.

As can be seen in Table 2, in the specific case (where five pies: *m* = 5 are allocated to four agents: *n* = 4), there are 225 different [P] _4×5_ matrices that ensure fairness within the NBS. For instance, if we follow a different partition set, as illustrated in Figure 3, i.e. the same partitions of the agent-coalitions into a pair for *n*-1 = 3 times, and a different partition of the pie-set *J* = {1,2,3,4,5} into two other subsets: {2,3} and {1,4,5}, then we calculate another [P] _4×5_ matrix:
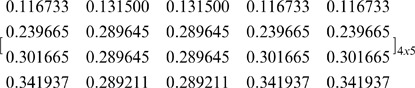
(31)


However, if we use this matrix along with the [*Π ^j^*]_ 5×1_,[C *_i_*]_ 4×1_ as inputs and the [*Π_i_*]_ 4×1_ as output in another MCS model, then the simulation gives: 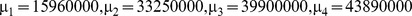 and 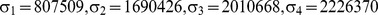, verifying that Eq (12) is also fulfilled with this [P] _4×5_ matrix.

**Figure 4:**
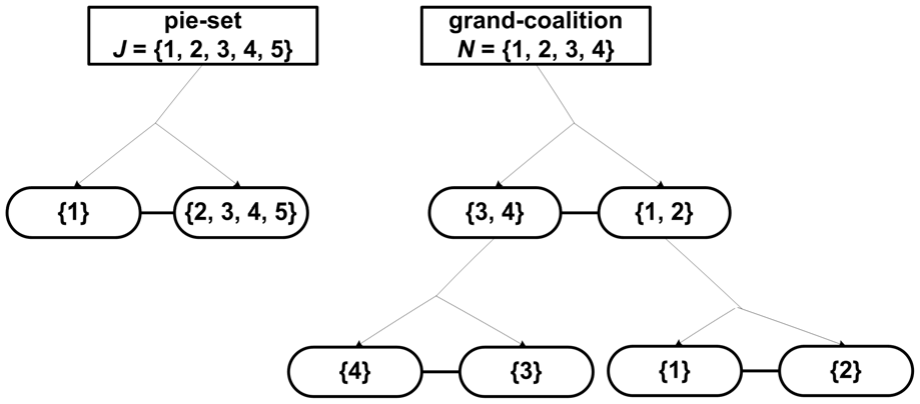
Third set of partitions.

Eq (12) can be also satisfied with the set of partitions presented in Figure 4. Specifically, through the partition of *J* = {1,2,3,4,5} into {1}, {2,3,4,5} subsets and the partition of *N* = {1,2,3,4} into {3,4}, {1,2}, and the further partitions into {4}, {3} and {1}, {2}, respectively, we get the following[P] _4×5_ matrix:
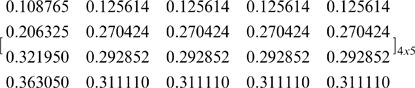
(32)with which the MCS gives:


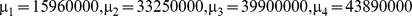
, and 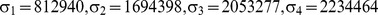.

Conclusively, it can be seen from Eqs (29), (31) and (32) that these [P] _4×5_ matrices are different, however provide equal results since the dividends allocated to all agents are distributed in proportion to the bargaining outcome U that is the asymmetric NBS with unequal divisions.

#### Asymmetric NBS with equal divisions

In the second case, the bargaining outcome is the asymmetric NBS where the surplus is divided equally according to Eq (6): U_1_ = U_2_ = U_3_ = U_4_ = 0.25. If we follow the same set of partitions presented in Figure 2, i.e. partition of *J* into two subsets: {1}, {2, 3, 4, 5} and partition of *N* into {1}, {2, 3, 4}, and the further partition into {4}, {2, 3}, and the further partition into {2}, {3}, then we compute the following [P] _4×5_ matrix:
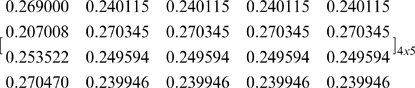
(33)


Moreover, if we develop another MCS model, where the estimated [P] _4×5_, the [*Π ^j^*]_ 5×1_ and the [C *_i_*]_ 4×1_ matrices are inputs and the [*Π _i_*]_ 4×1_ is the output in Eq (9), then the simulation gives: 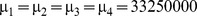, and 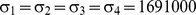. Clearly, this matrix ensures fairness within the asymmetric NBS with equal divisions, as it fulfils Eq (12):
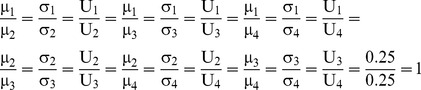



### Applications

In the previous numerical example, a generalized case of a cooperative venture was considered, in which agents with different disagreement payoffs agreed in a specific allocation of the overall surplus. However, there are various real life situations, to which the computation algorithm presented here can be applied. Specifically, it can be applied to all situations that can be formulated within Eq (9):
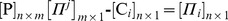
(9)


Indicatively, we present the following cases:

• Let’s consider a water allocation problem, in which *n* authorities (agents) with fixed disagreement payoffs of water (C_1_,…,C*_n_*), negotiate over the division of the water collected from different sources (pies), e.g. from rivers, rainwater tanks, wastewater treatment plants, etc. If these pies follow normal probability distributions and all agents are risk-neutrals and agree in a specific NBS (either symmetric or asymmetric), then the method presented here can be effectively applied. In particular, agents can compute a [P] *_n_*_×*m*_ matrix, which represents the ratio of the water from each source that should be distributed in each area, in order to ensure fairness within the NBS.
• Another example is a solid waste management problem, where the managers (agents) of *n* waste incineration plants, who have fixed disagreement payoffs of waste (C_1_,…,C*_n_*), negotiate over the division of the volume of solid waste (pies) arising from different areas. Specifically, if these pies follow normal probability distributions and the risk-neutral agents (who want to maximize the volume of waste treated in their plants, in order to maximize their profits) agree in a specific NBS, then the proposed algorithm can be applied for computation to the volume of waste that should be distributed in each plant from each area.
• Additionally, the computation algorithm can be applied to supply chain networks where *n* risk-neutral agents (manufacturers or/and suppliers or/and retailers), having fixed disagreement monetary payoffs (C_1_,…,C*_n_*), negotiate over the division of the revenues and costs that are yielded through cooperation. In this case, the pies represent the operation revenues, e.g. from different products’ selling, and the respective costs, e.g. infrastructure, advertising, distribution and storage costs, etc. Specifically, since the demand is not realized when the network is formed, these pies can be assumed as stochastic variables that follow normal probability distributions. If agents negotiate and agree in a specific NBS (either symmetric or asymmetric) for the division of the overall profits (surplus) of the network, then the proposed algorithm can be applied for computation to the ratio of each pie that should be allocated to each agent.

## Methods

We compute the possible [P] *_n_*_×*m*_ matrices that are presented in Table 2 by using the Wolfram Research Mathematica Version 7.0 software. For the development of MCS models within the computation algorithm, we suggest using specialized software, in order to get accurate results.

### Conclusions

Over the last decades, one of the most widely examined problems is the fair division of a surplus among agents. Herein, we focus on cases where multiple stochastic pies should be shared among agents with fixed disagreement payoffs, according to a specific NBS for the division of the overall surplus. Particularly, we consider that fairness is achieved when the stochastic dividends which are allocated to agents are distributed in proportion to the NBS. We introduce a novel method that can be applied to various situations, in order to compute the ratio of each pie that should be allocated to each agent. It is proved that there are finite possible solutions depending on the partitions of the pie-set into two subsets and the continuous partitions of the grand-coalition into two coalitions, until eventually all agents form singletons. We have assumed in this paper that all agents are risk-neutrals and the stochastic pies are divisible and follow normal probability distribution functions. Without making these assumptions, the development of respective algorithms in cases where agents with different risk preferences (risk-averse/neutral/seeking) are not indifferent between the *m* pies, or the stochastic pies are indivisible or follow asymmetric probability distributions, e.g. lognormal, can be subjects for future research. Future papers can also focus on the application of other solution concepts, such as the Shapley value, the core, and the Nucleolus to stochastic environments, where the size of the pie over which agents are negotiating may vary stochastically. Conclusively, the computation algorithm introduced with this paper can be a useful tool for decision-making under uncertainty, as it helps agents to estimate solutions that ensure fairness within a symmetric or asymmetric NBS.

## Supporting Information

Text S1 **Proofs of Theorems and Propositions.**
(DOC)
