# Supplementary material for: On the Fair Division of Multiple Stochastic Pies to Multiple Agents within the Nash Bargaining Solution
Source: PLoS One. 2012 Sep 14;7(9):e44535. doi: 10.1371/journal.pone.0044535 (PMC3443099; doi:10.1371/journal.pone.0044535)
Supplement: Text S1 — Proofs of Theorems and Propositions. (DOC) [file pone.0044535.s001.doc]

Proofs of Theorems and Propositions

**Proof of Theorem 1**. In order to ensure fairness for the division of the surplus *S* within the NBS, the stochastic dividends which are allocated to {1,…,*h*} and {*h*+1,…,*n*} coalitions, should be proportionally distributed, according to Eqs (10) and (11), i.e. to satisfy Eq (A.1):

$\frac{\text{μ}_{\left\{1,..,h\right\}}}{\text{μ}_{N}\sum_{i=1}^{h} \text{U}_{i}}\text{=}\frac{\text{μ}_{\left\{h+1,..n\right\}}}{\text{μ}_{N}\sum_{i=h+1}^{n} \text{U}_{i}}\Leftrightarrow\frac{\text{μ}_{\left\{1,..,h\right\}}}{\text{μ}_{\left\{h+1,..n\right\}}}\text{=}\frac{\sum_{i=1}^{h} \text{U}_{i}}{\sum_{i=h+1}^{n} \text{U}_{i}}$ and $\frac{\text{μ}_{\left\{1,..,h\right\}}}{\text{σ}_{\left\{1,..,h\right\}}}\text{=}\frac{\text{μ}_{\left\{h+1,..n\right\}}}{\text{σ}_{\left\{h+1,..n\right\}}}\Rightarrow$ $\frac{\text{μ}_{\left\{1,..,h\right\}}}{\text{μ}_{\left\{h+1,..n\right\}}}\text{=}\frac{\text{σ}_{\left\{1,..,h\right\}}}{\text{σ}_{\left\{h+1,..n\right\}}}=\frac{\sum_{i=1}^{h} \text{U}_{i}}{\sum_{i=h+1}^{n} \text{U}_{i}}$ (A.1)

However, the probability distribution functions of the {1,..,*h*} and {*h*+1,..,*n*} coalitions’ dividends, are given from Eqs (A.2) and (A.3), respectively:

$\Pi_{\left\{1,..,h\right\}}=S\sum_{i=1}^{h} \text{U}_{i}=\text{p}_{\left\{1,..,h\right\}}^{\left\{1,..,g\right\}}\sum_{j=1}^{g} \Pi^{j}+\text{p}_{\left\{1,..,h\right\}}^{\left\{g+1,..m\right\}}\sum_{j=g+1}^{m} \Pi^{j}\text{-}\sum_{i=1}^{h} \text{C}_{i}$ (A.2)

$\Pi_{\left\{h+1,..n\right\}}=S\sum_{i=h+1}^{n} \text{U}_{i}=\text{p}_{\left\{h+1,..n\right\}}^{\left\{1,..,g\right\}}\sum_{j=1}^{g} \Pi^{j}+\text{p}_{\left\{h+1,..n\right\}}^{\left\{g+1,..m\right\}}\sum_{j=g+1}^{m} \Pi^{j}\text{-}\sum_{i=h+1}^{n} \text{C}_{i}$ (A.3)

Taking into account that the efficient allocation of all pies is the only constraint considered:

$\text{p}_{\left\{1,..,h\right\}}^{\left\{1,..,g\right\}}+\text{p}_{\left\{h+1,..n\right\}}^{\left\{1,..,g\right\}}=\text{p}_{\left\{1,..,h\right\}}^{\left\{g+1,..m\right\}}+\text{p}_{\left\{h+1,..n\right\}}^{\left\{g+1,..m\right\}}=1$ (A.4)

i.e. the $\text{p}_{\left\{1,..,h\right\}}^{\left\{1,..,g\right\}},\text{p}_{\left\{h+1,..n\right\}}^{\left\{1,..,g\right\}},\text{p}_{\left\{1,..,h\right\}}^{\left\{g+1,..m\right\}},\text{p}_{\left\{h+1,..n\right\}}^{\left\{g+1,..m\right\}}$ can take any value with respect to Eq (A.4), we conclude that there is at least one [P] _2 x 2_ matrix: that satisfies Eq (A.1).

In order to prove the uniqueness of this solution, we assume that there is a second [P] _2 x 2_ matrix: , which also satisfies Eq (A.1). However, at least one element among the first and second matrices should be different: $\text{p}_{\left\{1,..,h\right\}}^{\left\{1,..,g\right\}}*\neq\text{p}_{\left\{1,..,h\right\}}^{\left\{1,..,g\right\}}**$, or $\text{p}_{\left\{h+1,..n\right\}}^{\left\{1,..,g\right\}}*\neq\text{p}_{\left\{h+1,..n\right\}}^{\left\{1,..,g\right\}}**$, or $\text{p}_{\left\{1,..,h\right\}}^{\left\{g+1,..m\right\}}*\neq\text{p}_{\left\{1,..,h\right\}}^{\left\{g+1,..m\right\}}**$, or $\text{p}_{\left\{h+1,..n\right\}}^{\left\{g+1,..m\right\}}*\neq\text{p}_{\left\{h+1,..n\right\}}^{\left\{g+1,..m\right\}}**$. We consider the following Eqs (A.5) and (A.6) for the pies’ mean values and Eqs (A.7) and (A.8) for all other values:

$\begin{matrix} \text{μ}_{\left\{1,..,h\right\}}=\text{μ}_{N}\sum_{i=1}^{h} \text{U}_{i}=\text{p}_{\left\{1,..,h\right\}}^{\left\{1,..,g\right\}}*\sum_{j=1}^{g} \text{μ}^{j}+\text{p}_{\left\{1,..,h\right\}}^{\left\{g+1,..m\right\}}*\sum_{j=g+1}^{m} \text{μ}^{j}\text{-}\sum_{i=1}^{h} \text{C}_{i}= \\ =\text{p}_{\left\{1,..,h\right\}}^{\left\{1,..,g\right\}}**\sum_{j=1}^{g} \text{μ}^{j}+\text{p}_{\left\{1,..,h\right\}}^{\left\{g+1,..m\right\}}**\sum_{j=g+1}^{m} \text{μ}^{j}\text{-}\sum_{i=1}^{h} \text{C}_{i} \end{matrix}$ (A.5)

$\begin{matrix} \text{μ}_{\left\{h+1,..n\right\}}=\text{μ}_{N}\sum_{i=h+1}^{n} \text{U}_{i}=\text{p}_{\left\{h+1,..n\right\}}^{\left\{1,..,g\right\}}*\sum_{j=1}^{g} \text{μ}^{j}+\text{p}_{\left\{h+1,..n\right\}}^{\left\{g+1,..m\right\}}*\sum_{j=g+1}^{m} \text{μ}^{j}\text{-}\sum_{i=h+1}^{n} \text{C}_{i}= \\ =\text{p}_{\left\{h+1,..n\right\}}^{\left\{1,..,g\right\}}**\sum_{j=1}^{g} \text{μ}^{j}+\text{p}_{\left\{h+1,..n\right\}}^{\left\{g+1,..m\right\}}**\sum_{j=g+1}^{m} \text{μ}^{j}\text{-}\sum_{i=h+1}^{n} \text{C}_{i} \end{matrix}$ (A.6)

$\begin{matrix} \Pi_{\left\{1,..,h\right\}}=S\sum_{i=1}^{h} \text{U}_{i}=\text{p}_{\left\{1,..,h\right\}}^{\left\{1,..,g\right\}}*\sum_{j=1}^{g} \Pi^{j}+\text{p}_{\left\{1,..,h\right\}}^{\left\{g+1,..m\right\}}*\sum_{j=g+1}^{m} \Pi^{j}\text{-}\sum_{i=1}^{h} \text{C}_{i}= \\ =\text{p}_{\left\{1,..,h\right\}}^{\left\{1,..,g\right\}}**\sum_{j=1}^{g} \Pi^{j}+\text{p}_{\left\{1,..,h\right\}}^{\left\{g+1,..m\right\}}**\sum_{j=g+1}^{m} \Pi^{j}\text{-}\sum_{i=1}^{h} \text{C}_{i} \end{matrix}$ (A.7)

$\begin{matrix} \Pi_{\left\{h+1,..n\right\}}=S\sum_{i=h+1}^{n} \text{U}_{i}=\text{p}_{\left\{h+1,..n\right\}}^{\left\{1,..,g\right\}}*\sum_{j=1}^{g} \Pi^{j}+\text{p}_{\left\{h+1,..n\right\}}^{\left\{g+1,..m\right\}}*\sum_{j=g+1}^{m} \Pi^{j}\text{-}\sum_{i=h+1}^{n} \text{C}_{i}= \\ =\text{p}_{\left\{h+1,..n\right\}}^{\left\{1,..,g\right\}}**\sum_{j=1}^{g} \Pi^{j}+\text{p}_{\left\{h+1,..n\right\}}^{\left\{g+1,..m\right\}}**\sum_{j=g+1}^{m} \Pi^{j}\text{-}\sum_{i=h+1}^{n} \text{C}_{i} \end{matrix}$ (A.8)

However, from Eqs (A.5) to (A.8), we get:

$(\text{p}_{\left\{1,..,h\right\}}^{\left\{1,..,g\right\}}*\text{-p}_{\left\{1,..,h\right\}}^{\left\{1,..,g\right\}}**)\sum_{j=1}^{g} \text{μ}^{j}+(\text{p}_{\left\{1,..,h\right\}}^{\left\{g+1,..m\right\}}*\text{-p}_{\left\{1,..,h\right\}}^{\left\{g+1,..m\right\}}**)\sum_{j=g+1}^{m} \text{μ}^{j}=0$ (A.9)

$(\text{p}_{\left\{h+1,..n\right\}}^{\left\{1,..,g\right\}}*\text{-p}_{\left\{h+1,..n\right\}}^{\left\{1,..,g\right\}}**)\sum_{j=1}^{g} \text{μ}^{j}+(\text{p}_{\left\{h+1,..n\right\}}^{\left\{g+1,..m\right\}}*\text{-p}_{\left\{h+1,..n\right\}}^{\left\{g+1,..m\right\}}**)\sum_{j=g+1}^{m} \text{μ}^{j}=0$ (A.10)

$(\text{p}_{\left\{1,..,h\right\}}^{\left\{1,..,g\right\}}*\text{-p}_{\left\{1,..,h\right\}}^{\left\{1,..,g\right\}}**)\sum_{j=1}^{g} \Pi^{j}+(\text{p}_{\left\{1,..,h\right\}}^{\left\{g+1,..m\right\}}*\text{-p}_{\left\{1,..,h\right\}}^{\left\{g+1,..m\right\}}**)\sum_{j=g+1}^{m} \Pi^{j}=0$ (A.11)

$(\text{p}_{\left\{h+1,..n\right\}}^{\left\{1,..,g\right\}}*\text{-p}_{\left\{h+1,..n\right\}}^{\left\{1,..,g\right\}}**)\sum_{j=1}^{g} \Pi^{j}+(\text{p}_{\left\{h+1,..n\right\}}^{\left\{g+1,..m\right\}}*\text{-p}_{\left\{h+1,..n\right\}}^{\left\{g+1,..m\right\}}**)\sum_{j=g+1}^{m} \Pi^{j}=0$ (A.12)

and by summing Eq (A.9) with (A.11) and Eq (A.10) with (A.12), we have:

$\begin{matrix} (\text{p}_{\left\{1,..,h\right\}}^{\left\{1,..,g\right\}}*\text{-p}_{\left\{1,..,h\right\}}^{\left\{1,..,g\right\}}**)(\sum_{j=1}^{g} \text{μ}^{j}+\sum_{j=1}^{g} \Pi^{j}) \\ +(\text{p}_{\left\{1,..,h\right\}}^{\left\{g+1,..m\right\}}*\text{-p}_{\left\{1,..,h\right\}}^{\left\{g+1,..m\right\}}**)(\sum_{j=g+1}^{m} \text{μ}^{j}+\sum_{j=g+1}^{m} \Pi^{j})=0 \end{matrix}$ (A.13)

$\begin{matrix} (\text{p}_{\left\{h+1,..n\right\}}^{\left\{1,..,g\right\}}*\text{-p}_{\left\{h+1,..n\right\}}^{\left\{1,..,g\right\}}**)(\sum_{j=1}^{g} \text{μ}^{j}+\sum_{j=1}^{g} \Pi^{j}) \\ +(\text{p}_{\left\{h+1,..n\right\}}^{\left\{g+1,..m\right\}}*\text{-p}_{\left\{h+1,..n\right\}}^{\left\{g+1,..m\right\}}**)(\sum_{j=g+1}^{m} \text{μ}^{j}+\sum_{j=g+1}^{m} \Pi^{j})=0 \end{matrix}$ (A.14)

Due to the fact that in both Eqs (A.13), (A.14), the: $(\sum_{j=1}^{g} \text{μ}^{j}+\sum_{j=1}^{g} \Pi^{j})>0$, and $(\sum_{j=g+1}^{m} \text{μ}^{j}+\sum_{j=g+1}^{m} \Pi^{j})>0$, it follows immediately that both the 1^st^ and 3^rd^ parentheses in each Eq should equal zero, hence:

$\begin{matrix} \text{p}_{\left\{1,..,h\right\}}^{\left\{1,..,g\right\}}*=\text{p}_{\left\{1,..,h\right\}}^{\left\{1,..,g\right\}}**,\text{ p}_{\left\{1,..,h\right\}}^{\left\{1,..,g\right\}}*=\text{p}_{\left\{1,..,h\right\}}^{\left\{1,..,g\right\}}**,\text{ } \\ \text{p}_{\left\{1,..,h\right\}}^{\left\{g+1,..m\right\}}*=\text{p}_{\left\{1,..,h\right\}}^{\left\{g+1,..m\right\}}**,\text{ p}_{\left\{h+1,..n\right\}}^{\left\{g+1,..m\right\}}*=\text{p}_{\left\{h+1,..n\right\}}^{\left\{g+1,..m\right\}}** \end{matrix}$ (A.15)

Therefore, the second matrix equals the first and thus, there is a unique [P] _2 x 2_ matrix for each two pairs of non-empty coalitions and subsets that can arise from the partitions of the grand-coalition *N* and the pie-set *J*.

**Proof of Proposition 1**. In the partition of the pie-set *J* = {1,..,*m*} into two non-empty subsets {1,..,*g*} and {*g*+1,..,*m*}, there is no constraint considered for the *m* ≥ 2 pies, i.e. any pie can be placed either in the first or in the second subset, in which the order of pies does not matter. Particularly, there are: *m*!/(*m*-1)! combinations for a pair with one-pie and (*m*-1) pies, respectively. However, if *m* = odd, then the number of combinations in subsets of: 2-pies and (*m*-2)-pies, 3-pies and (*m*-3)-pies, ..., is given from: *m*!/(*m*-*l*)!*l*!, where *l* = 3,5,7,..., (*m*-1)/2. On the other hand, the respective combinations if *m* = even, is given from: *m*!/(*m*-*l*)!*l*! +m!/2((m/2)!)^2^, i.e. for *l* = 4,6,8,..., (*m*/2)-1.

Hence, the number of possible pairs of non-empty subsets that can arise from the partition of pie-set *J*, is given from the piecewise Eq (18).

**Proof of Theorem 2**. From Proposition 1 we get Eq (19.2), while the same combinations hold for the partition of the grand-coalition into two non-empty coalitions. However, in the partition of a set: *N* = {1,..,*n*} into two coalitions*:* {1,.., *h*} and {*h*+1,..,*n*}, the number of possible solutions provided by each partition equals the respective possible solutions included in each coalition.

- For instance, when *n* =2, i.e. *N* = {1,2}, there is only one partition of the *N* that is {1} and {2}. Therefore, from Theorem 1 there is a unique [P] _2 x 2_ matrix: , and the *f*(2) = 1.
- Moreover, when *n* = 3, i.e. *N* = {1,2,3}, there are 3 possible partitions into a pair of 1-agent and 2-agent coalitions:

### *i. {1} and {2,3} and the further partition into {2}and {3}, gives a first* [P] _3 x 2_ *matrix:*

### *{2} and {1,3} and the further partition into {1} and {3} gives a second* [P]_3 x 2_ *matrix*:

### *{3} and {1,2} and the further partition into {1} and {2} gives a third* [P] _3 x 2_ *matrix*:

Therefore *f*(3) = 3.

- - - - For *N* = {1,2,3,4}, there are 4!/(3!(4-3)!)=4 possible partitions into a pair of 1-agent and 3-agent coalitions:

1. {1,2,3} and {4},
2. {1,2,4} and {3},
3. {1,3,4} and {2},
4. {2,3,4} and {1},

where each partition includes 3 possible solutions (for the further partitions of the 3-agent coalitions). Furthermore, there are also three possible partitions of the *N* into a pair of 2-agent and 2-agent coalitions:

1. {1,2} and {3,4} ,
2. {1,3} and {2,4},
3. {1,4} and {2,3},

where each one includes 1 solution (for the further partitions of the 2-agent coalitions). Therefore:$f(4)=4f(3)+3f\text{(2)}=15$

- - - - For *n* ≥ 5, the number of possible solutions provided by the continuous partitions of all agent-coalitions into two nonempty coalitions, is computed through Eq (19.1).

Taking into account that any partition of the pie-set can be combined with any partition of the agent-coalition into two nonempty coalitions, we conclude that the total number of possible solutions, ([P] *_n X m_* matrices) is given from Eq (19): *f*(*n*)*g*(*m*)

However, it can be seen in Eq (19) that the number of possible solutions depends on the number of pies *m* and the number of agents *n*. Due to the fact that both *N*, *J* are finite sets, we conclude that there are finite [P] *_n X m_* matrices, which ensure that the stochastic dividends which are allocated to agents are distributed in proportion (with fairness) to the NBS.
